# A Rapid Molecular Approach for Chromosomal Phasing

**DOI:** 10.1371/journal.pone.0118270

**Published:** 2015-03-04

**Authors:** John F. Regan, Nolan Kamitaki, Tina Legler, Samantha Cooper, Niels Klitgord, George Karlin-Neumann, Catherine Wong, Shawn Hodges, Ryan Koehler, Svilen Tzonev, Steven A. McCarroll

**Affiliations:** 1 Digital Biology Center, Bio-Rad Laboratories, Pleasanton, California, United States of America; 2 Department of Genetics, Harvard Medical School, Boston, Massachusetts, United States of America; 3 Program in Medical and Population Genetics and Stanley Center for Psychiatric Research, Cambridge, Massachusetts, United States of America; Johns Hopkins University, UNITED STATES

## Abstract

Determining the chromosomal phase of pairs of sequence variants – the arrangement of specific alleles as haplotypes – is a routine challenge in molecular genetics. Here we describe Drop-Phase, a molecular method for quickly ascertaining the phase of pairs of DNA sequence variants (separated by 1-200 kb) without cloning or manual single-molecule dilution. In each Drop-Phase reaction, genomic DNA segments are isolated in tens of thousands of nanoliter-sized droplets together with allele-specific fluorescence probes, in a single reaction well. Physically linked alleles partition into the same droplets, revealing their chromosomal phase in the co-distribution of fluorophores across droplets. We demonstrated the accuracy of this method by phasing members of trios (revealing 100% concordance with inheritance information), and demonstrate a common clinical application by phasing *CFTR* alleles at genomic distances of 11–116 kb in the genomes of cystic fibrosis patients. Drop-Phase is rapid (requiring less than 4 hours), scalable (to hundreds of samples), and effective at long genomic distances (200 kb).

## Introduction

Sequencing and genotyping identify the alleles that are present in a diploid genome without revealing their arrangement as haplotypes. Knowing the chromosomal phase of genomic sequence variants is often important for genetic analysis and for fully exploiting the potential of techniques such as genome engineering and allele-specific expression analysis.

We briefly describe four genetics research scenarios, among many others, in which phase information is important. (i) *Compound heterozygosity*. Whenever a gene has multiple deleterious alleles in the same individual, or in a cancer, determining whether such alleles are present on the same chromosomal copy of the gene (in *cis*) or the opposite copy (in *trans*, potentially inactivating both copies) is central to genetic interpretation. (ii) *Allele-specific expression analysis* provides precise ways of measuring the effects of cis-acting regulatory variants on nearby genes [1,2]—but its effective use requires information about chromosomal phase to evaluate the direction of effect (increased or decreased expression) of regulatory variants. (iii) *Parent-of-origin* analysis of new mutations is important in genetic counseling and in research about male and female mutation rates and effects of paternal age; such analysis is today often limited to research scenarios in which three generations can be sequenced [3,4], or to the small subset of mutations that are near inherited variants [5]. (iv) *Genome engineering* is beginning to attain widespread use as a way to evaluate the functional consequence of genome variants [6–9]. In humans and other species with extensive heterozygosity, it will often be important to know the chromosomal phase of experimenter-made genome edits, which affect a random chromosomal copy, with respect to the rare and common functional variants that are already present at a locus of interest.

For rare variants, new mutations, and genome edits, chromosomal phase cannot be inferred by population-based statistical methods; even for common polymorphisms, statistical inference of phase is only probabilistically accurate. Family-based data are useful for phasing, but are available only in select contexts. Thus, molecular methods for phasing have been important in both research and clinical applications.

Existing molecular methods for phasing pairs of variants involve long-range PCR, cloning, and/or manual dilution to single-molecule concentrations. An important class of methods has involved single-molecule dilution (SMD) [10] which can be followed by PCR and mass spectrometry [11], or by amplification and Sanger sequencing [12]^,^[13]. SMD is quite effective, though SMD requires manual dilutions of DNA samples to single-molecule concentrations. Another class of methods involves long-range PCR, which can be combined with intra-molecular ligation [14], or use allele-specific primers [15,16], or be followed by cloning and Sanger sequencing, to detect linked alleles. Long-range PCR can also be successful, though is limited to the scale of PCR amplicons (generally < 20 kb). When the value of genome-wide information justifies the investments in constructing clone libraries, libraries can be constructed and subjected to high-throughput sequencing in barcoded pools [17]. Cloning-free methods utilizing SMD (dilution of genomic DNA into sub-haploid quantities across many individual wells) followed by multiple displacement amplification, barcoding, and NGS have also recently been described [18,19].

The day-to-day use of molecular phasing approaches has been limited by cost and time requirements (cloning, manual limiting dilution) or genomic range (PCR). A key need is for fast, low-cost approaches that a scientist could apply in an afternoon and to many samples at once.

Recent innovations in microfluidics allow biochemical reactions to be quickly partitioned into thousands of nanoliter-sized droplets (aqueous compartments in an oil-aqueous emulsion) and allow fluorescence signals in such droplets to be quickly quantified [20]; devices for making and analyzing droplets are now available in many research labs. Droplets allow single-molecule dilution to be accomplished within individual reaction vessels (wells), a feature which we hypothesized could be combined with allele- fluorescence probes (from pairs of loci) and customized statistical analysis methods to support rapid, inexpensive molecular phasing.

Here we describe Drop-Phase, a method for rapidly phasing pairs of genomic sequences in sets of 1 to 96 genomes, at low cost and in a few hours’ work.

## Material and Methods

### Digital droplet PCR

Droplet digital PCR (ddPCR) involves the use of readily generated oil/aqueous reverse emulsions to partition a reaction into thousands of tiny, nanoliter-volume reaction compartments [20]. Microfluidics support the creation of monodisperse emulsions in which droplets have a uniform volume [20]; such emulsions are created in about two minutes (per reaction) using “droplet generation” devices that are now available in many research labs (Bio-Rad Laboratories, Hercules, CA, USA). We performed ddPCR as described in earlier studies [20], with a few important differences. First, we used wide-bore pipette tips during gDNA manipulations, and used gentle reaction mixing to preserve the longer fragments present in gDNA samples. Second, in some experiments in which longer-range (>30 kb) phasing was desired, we extracted the DNA using methods (described below) that maximize the yield of long fragments. Finally, in contrast to standard ddPCR analysis, in which gDNA is digested into smaller fragments using a restriction enzyme, we used undigested DNA (except in control experiments, as described below).

For this study, droplet digital reactions consisted of gDNA, FAM, and HEX fluorescent hydrolysis probe assays and ddPCR Supermix for Probes (no dUTP)(Bio-Rad). gDNA was added to the reactions at ~650 pg/μL (200 human haploid targets/μL) as determined by *A*_260_ measurements from a NanoDrop 8000 spectrophotometer (Thermo Scientific, Waltham, MA). The Bio-Rad droplet generator emulsifies reaction mixtures into 0.85 nL droplets, which were transferred into 96 well plates (Eppendorf, Hamburg, DE), sealed with a pierceable foil heat seal (Bio-Rad), and cycled in a C1000 thermal cycler (Bio-Rad) using one of the following two protocols: 1) 95°C for 10 min (1 cycle), (94°C for 30 s, 60°C for 1 min) for 40 cycles, 98°C for 10 min (1 cycle) or 2) 95°C for 10 min (1 cycle), (94°C for 30 s, 55°C for 1 min) for 40 cycles, 98°C for 10 min (1 cycle), for the “mile marker” and *CFTR* phasing experiments, respectively. Ramp rates were set to 2.0°C/s. The droplets were read using a QX200 droplet reader and data analyzed using QuantaSoft v1.4.0.99 (Bio-Rad). The QuantaSoft software contains an embedded table that includes columns for ‘concentration’ (total concentration of targeted sequences) and ‘linkage’ (concentration of linked sequences), which are reported in copies/μL.

### Samples

All cell lines and DNA samples were obtained from the Coriell Institute for Medical Research under an approved material transfer agreement (MTA) and assurance form. Sample GM18916 is an Epstein-Barr virus transformed B-lymphocyte cell line from the Yoruba in Ibadan, Nigeria and was part of the International HapMap Project [21], and was used for the mile marker experiment. This cell line was passaged in Roswell Park Memorial Institute medium 1640 supplemented with 2 mol/m^3^ L-glutamine (Sigma–Aldrich, St. Louis, MO, USA) and 15% fetal bovine serum (Corning Inc., Corning, NY, USA).

The cystic fibrosis cell lines derived from Epstein-Barr virus transformed B-lymphocytes included: GM11286 and GM11274, which were determined to have c.1652G>A (p.Gly551Asp) and c.1521_1523delCTT (p.Phe508del) variants [22]; GM11279, which was determined to have 129G>C (promoter), c.350G>A (p.Arg117His), and c.1521_1523delCTT (p.Phe508del) variants [23]; GM11472, which was characterized to have c.1210–12T[7], c.1210–12T[9], c.3909C>G (p.Asn1303Lys), and c.4046G>A (p.Gly1349Asp) variants [24,25] (c.4046G>A is also referred to as c.4178G>A in some dbSNP databases); and GM13591, which was characterized to have c.350G>A (p.Arg117His), c.1210–12T[5], c.1210–12T[9], and c.1521_1523delCTT (p.Phe508del) variants [26]. These cystic fibrosis cell lines were propagated in the same medium as GM18916.

One untransformed fibroblast cell line, GM03465, was included in the study, and was characterized to have c.1652G>A (p.Gly551Asp) and c.1521_1523delCTT (p.Phe508del) variants [22]. This cell line was passaged in Eagle's Minimum Essential Medium with Earle's salts supplemented with nonessential amino acids (Sigma—Aldrich), 2 m*M* L-glutamine (Sigma–Aldrich), and 15% fetal bovine serum (Corning Inc.).

### Assays

The assays used in the mile marker and CFTR phasing experiments are described in S1 Table and S2 Table, respectively. All primers and Iowa Black quenched probes (IABkFQ) were ordered from Integrated DNA Technologies (Coralville, IA, USA), whereas all TaqMan-MGB probes were ordered from Life Technologies (Carlsbad, CA, USA). The targeted residue(s) of interest is shown as a lowercase letter. The concentrations of the assay components were 900 n*M*, 250 n*M*, and 1000 n*M* for primers, probe, and dark probe, respectively. Non-fluorescent (“dark”) competitor probes were only used in the phasing assays to reduce cross-reactivity with the non-targeted allele.

### Sample extraction

GM18916 cells were pelleted at 250 × *g* for 5 min, washed with 1X phosphate buffered saline (PBS), pelleted again at 250 × *g* for 5 min, and resuspended in 1X PBS to a final concentration of approximately 7 × 10^6^ cells/mL as measured by TC10 Automated Cell Counter (Bio-Rad). The cells were split into 40 μL aliquots, each with a total cell count of approximately 2.8 × 10^5^ cells. Cells were processed for DNA extraction using either polysaccharide precipitation–based chemistry (PrepFiler Forensic DNA Extraction Kit, Life Technologies) or silica column–based chemistry (DNeasy Blood and Tissue Kit, Qiagen, Valencia, CA, USE).

### Polysaccharide precipitation

A 40 μL sample of 7 × 10^6^ cells/mL in 1× PBS was extracted using the PrepFiler Forensic DNA Extraction Kit (Life Technologies). The manufacturer’s recommended protocol was used, with the following exceptions: sample lysis incubation was reduced from the recommended 20 min at 70°C to 10 min with no shaking of the sample during incubation; any mixing and/or washing of the samples by vortexing was replaced with gentle end-over-end inversion except for the mixing step immediately following the addition of 180 μL isopropyl alcohol, which was performed on the lowest rpm setting of a vortex mixer; centrifugation of samples was kept to brief spins only; and any transfer of sample after cell lysis was performed using only wide-bore pipette tips (Rainin, a Mettler Toledo company, Oakland, CA, USA). DNA was eluted in 50 μL of kit-provided elution buffer.

### Silica columns

A 40 μL sample of 7 × 10^6^ cells/mL in 1× PBS was extracted using the DNeasy Blood and Tissue Kit (Qiagen). The manufacturer’s recommended protocol was used with the following exceptions: 160 μL of 1× PBS was added to the 40 μL sample to bring the sample volume to 200 μL before adding 200 μL of buffer AL with proteinase K; after adding buffer AL with proteinase K, the sample was mixed with gentle end-over-end inversion; and any transfer of the sample after cell lysis was performed using wide-bore pipette tips (Rainin). DNA was eluted in 200 μL of AE buffer.

### Calculation of linkage

**Fig 1:**
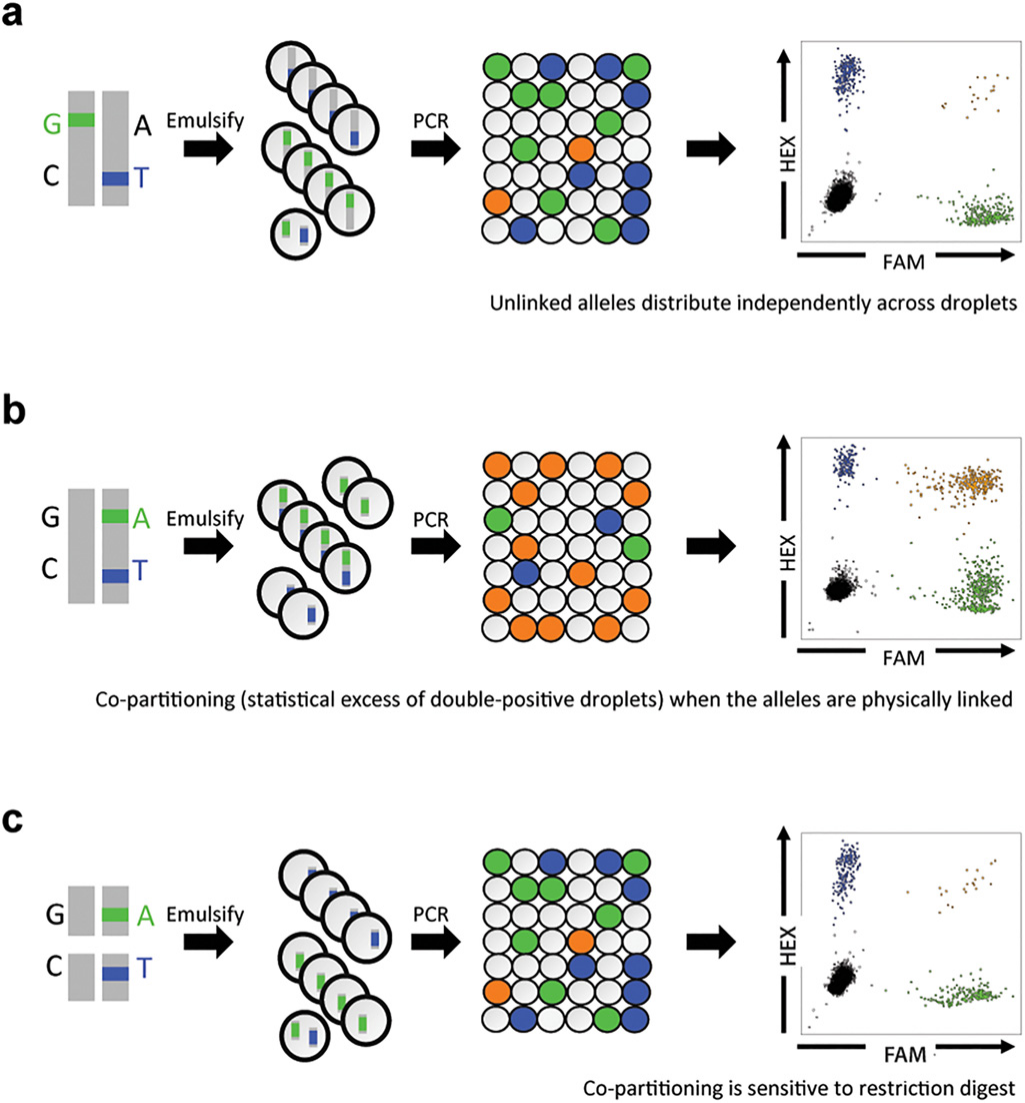
Drop-Phase schematic. A genomic DNA sample is emulsified into aqueous droplets in an oil-aqueous reverse emulsion. Allele-specific fluorescence probes (FAM, blue; and HEX, green) are used to detect alleles at two different loci. Following PCR, the droplets are positive for one fluorophore (blue or green), positive for both fluorophores (orange), or positive for neither fluorophore, depending on the alleles they contained at the beginning of the reaction. (**a**) *Trans*-configured alleles partition independently into droplets. Co-partitioning (orange) is therefore governed by chance. (**b**) *Cis*-configured alleles tend to co-segregate into the same droplets, because they are physically linked; co-partitioning greatly exceeds chance expectation. (**c**) Restriction digest at a site between the *cis*-configured alleles abolished co-partitioning of the two alleles; co-partitioning again occurs to the extent expected by chance.

Detection of linkage is based on the observation that presence of linked DNA molecules will increase the number of double-positive droplets relative to the number expected due to chance (Fig. 1). We describe the mathematical calculation of linkage in S1 Note. Linkage can be measured in absolute terms (the absolute concentration of linked molecules) or in relative terms (the percent of all molecules that are linked). In this manuscript, we focus on the percent of all molecules that are linked, as this measurement is not affected by DNA input concentration and is therefore a property of the DNA sample under analysis.

### Controls for determining linkage

**Fig 2:**
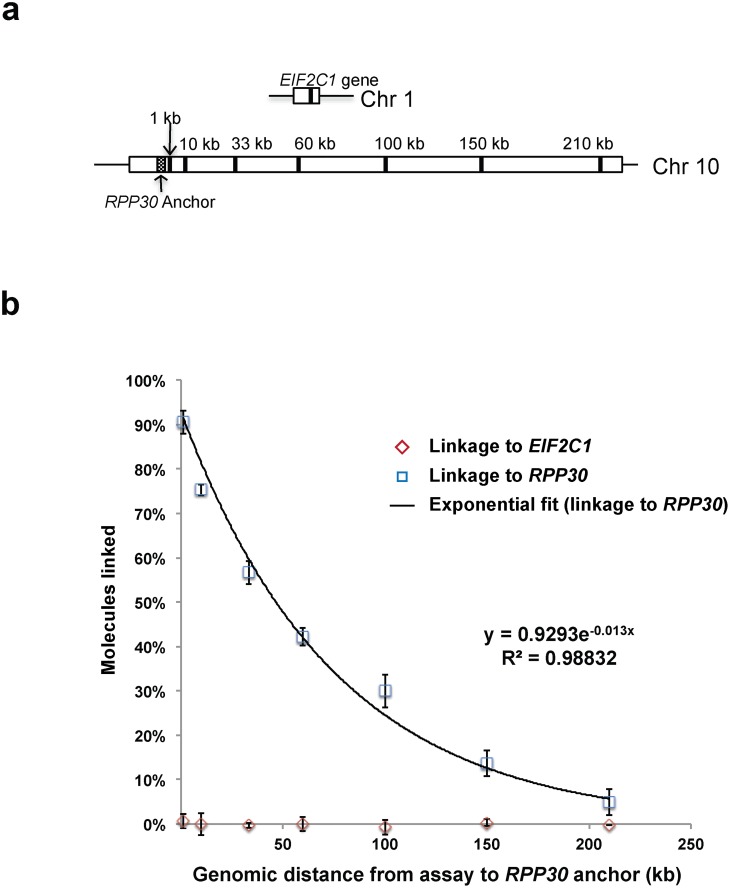
Evaluation of the relationship of physical linkage to genomic distance, using polysaccharide precipitation-extracted DNA. (**a**) In this analysis, FAM-labeled “mile marker” assays targeting sequences at different distances (1–210 kb) from the *RPP30* anchor sequence were paired with a HEX-labeled assay specific to the *RPP30* anchor sequence. Control assays utilized an anchor assay sequence in *EIF2C*, which resides on another chromosome. (**b**) The percentage of linked molecules at each genomic distance is shown as a function of distance. Means (of triplicate measurements) and 95% confidence intervals are shown.

We utilized three different strategies for negative controls depending on the experiment. For the experiment in which we confirmed the phase of variants that had been inferred from inheritance data, we used restriction enzymes to specifically cut the DNA sequences between the heterozygous SNPs being phased. In the *CFTR* phasing experiment, we assembled four unique duplexes to cover all possible combinations for a pair of heterozygous SNPs. By design, given sufficiently intact DNA, two of the duplexes will be linked and provide the diplotype of the region, whereas the other two will not be linked (negative control). Failure to measure a difference in the percentage of linked molecules between the linked (n = 2) and unlinked (n = 2) duplexes suggests the DNA between the loci is too fragmented to confirm the phase of the region. In the “mile marker” analysis of linkage as a function of genomic distance (Fig. 2) the negative controls were comprised of duplex assays in which individual mile marker assays were paired with an assay targeting the *EIF2C1* gene on a different chromosome (chromosome 1).

## Results

### Drop-Phase measures the co-partitioning of DNA sequences into droplets

Drop-Phase utilizes droplet digital PCR (ddPCR), which involves subdividing a reaction mixture into thousands of nanoliter-sized aqueous droplets in an oil-aqueous emulsion, amplifying DNA within the droplets, and then counting the droplets that contain the product of interest [20,27,28]. Microfluidic devices for droplet generation and analysis are widely used today [29–31]. To simultaneously evaluate the presence of two sequences of interest—for example, two SNP alleles at different loci—we use multiple fluorescence reporters (e.g. FAM and HEX fluorophores). Drop-Phase determines genomic phase by analyzing the extent to which alleles at two different genomic loci reside in the same droplets. Our approach is based on a simple idea: when two alleles are physically linked, they tend to partition into the same droplets.

In each experiment, a genomic DNA sample (10–20 ng) is partitioned into about 20,000 aqueous droplets in an oil/aqueous reverse emulsion (<2 minutes); both loci are amplified within the droplets in the presence of allele-specific fluorescence probes that report on the presence of alleles of interest (<90 minutes); and the resultant fluorescence signals are detected in each droplet (<2.5 minutes per sample).

### Drop-Phase results are consistent with inheritance

We first performed a simple proof-of-concept experiment by chromosomally phasing four pairs of heterozygous SNPs in father-mother-offspring trios for whom the phases of these SNPs could be independently established by inheritance. In experiments in which the fluorescent probes detected alleles that resided on different chromosomal copies (“*trans*-configured” alleles, as established by inheritance from different parents), the fluorescence signals were distributed independently across droplets, co-localizing only to the extent expected by chance (*p* > 0.1 by chi-square test in each case; Fig. 1a). When the fluorescence probes detected alleles that resided on the same chromosomal copy (“*cis*-configured” alleles, established by inheritance from the same parent), the number of droplets positive for both fluorophores greatly exceeded chance expectation (*p* < 10^–16^ in each case; Fig. 1b). For SNPs at genomic distances of a few kilobases, most droplets that were positive for one fluorophore were positive for both fluorophores.

To confirm that this enrichment of double-positive droplets was due to physical linkage of the alleles, we digested the DNA with a restriction enzyme specific to a site between the two loci before distributing the genomic DNA into droplets (Fig. 1c). Digestion greatly reduced the frequency of double-positive droplets relative to the undigested sample (*p* < 10^–6^) (Fig. 1c). This result confirmed that the co-partitioning in the earlier experiment (Fig. 1b) was due to physical linkage of the SNP alleles (Fig. 1c).

Similarly definitive and accurate determinations of chromosomal phase were achieved for four pairs of heterozygous SNPs (spanning 1–40 kb) assayed in seven individuals in 100% of assays (14/14). These data agreed with the prediction from inheritance, indicating that at least at modest genomic distances (1–40 kb), Drop-Phase can quickly and reliably phase pairs of SNPs.

### The physical range of analysis is limited primarily by DNA fragment size

We next sought to evaluate the genomic distance at which physical linkage or chromosomal phase can be established by Drop-Phase. Because the extraction of genomic DNA causes chromosomes to fragment into smaller pieces, most genomic DNA samples contain DNA segments of various sizes. Even when two DNA sequences (alleles) are physically linked in the proband’s genome, those sequences will be physically linked on only some of the DNA fragments under analysis. The greater the genomic distance separating the DNA sequences, the smaller the fraction of DNA fragments that will contain both sequences. It was therefore important to understand the mathematical and empirical relationships among genomic distance, DNA fragmentation, and co-partitioning in droplets.

DNA fragmentation limits the extent to which cis-configured sequences will co-localize. The more fragmented a DNA sample is, the smaller the fraction of DNA molecules that will contain both sequences in a physically linked form, and the larger the fraction that will bear the sequences individually. For any specific DNA sample, we define *%linkage* as the percentage of all DNA molecules containing sequence *A* that also contain sequence *B*. If *A* and *B* are unlinked in the donor’s genome—for example, if they are alleles that are trans-configured (on different chromosomal copies)—then *%linkage* should be zero. We derived a mathematical formula for estimating *%linkage* from the numbers of (*A+B+*), (*A+B-*), (*A-B+*), and (*A-B-*) droplets in a Drop-Phase experiment (S1 Note, S1 Fig.). Note that when *%linkage* is very small, a sample in which two alleles are *cis*-configured may become indistinguishable from a sample in which the alleles are *trans*-configured. This scenario defines the detection limit of Drop-Phase.

To understand empirically the limitations of linkage and phasing analysis in droplets, we designed assays to measure *%linkage* at a series of genomic distances (1, 10, 33, 60, 100, 150, and 210 kb) from a fixed marker (Fig. 2a). (For this experiment, we utilized non-polymorphic sequences, since our goal was not to phase but simply to measure the physical intactness of genomic DNA in a simple way.) We then used these assays to evaluate *%linkage* as a function of distance in genomic DNA samples isolated by two common approaches: silica-based column (DNeasy, Qiagen) and polysaccharide-based precipitation onto magnetic particles (PrepFiler, Life Technologies). In genomic DNA derived by the polysaccharide-based precipitation, linkage was readily recognized at all distances tested: at 60 kb, *%linkage* was approximately 42%; even at 210 kb, *%linkage* was approximately 5% and still clearly distinguishable from control analyses of unlinked loci from different chromosomes (Fig. 2a,b; *p* < 10^–20^ at each tested distance for the linked loci; *p* > 0.1 at each tested distance for unlinked loci, by Pearson chi-square test). By contrast, in DNA samples derived from silica-based columns, linkage could be reliably detected only out to about 60 kb (S2 Fig.). Thus, droplets can be used to analyze linkage and phase at substantial genomic distances even when DNA is extracted using conventional kit-based strategies. (Unconventional extraction methods might allow analysis at even longer genomic distances, but our emphasis here is on easy, scalable methods.)

### Drop-Phase in an example application: compound heterozygosity

We next sought to evaluate the utility and efficacy of this approach in a common, real-world application: ascertaining the chromosomal phase of deleterious variants across the *CFTR* gene, which spans 189 kb. Recessive, non-complementing alleles of *CFTR* are the most common cause of congenital genetic illness in populations with European ancestry. Individuals with one compromised *CFTR* copy are carriers; individuals with both *CFTR* copies compromised have cystic fibrosis [32]. Nearly 2,000 variants have been described in *CFTR*, of which 127 (mostly rare) variants are thought to be pathogenic [33]. Strongly compromised *CFTR* alleles include ΔF508 (one of the first Mendelian recessive variants discovered by positional cloning [34–36]) and c.1652G>A; these variants are pathogenic when *trans*-configured, as this arrangement leaves an individual with no functional copy of the gene. About 3% of individuals with European ancestry are carriers of ΔF508, which therefore frequently appears in individuals and families together with other *CFTR* variants [33]. Some milder *CFTR* variants are benign when present alone, but when arranged in *cis* with other such variants, can form a more compromised haplotype that fails to complement the more common ΔF508 allele [37,38]. An example of a compromised haplotype of *CFTR* involves the combination of the c.350G>A protein-coding variant with the intronic c.1210–12T[5] repeat expansion polymorphism [37]. Understanding how these and other variants are arranged onto haplotypes is important for diagnostics and preconception carrier screening.

**Fig 3:**
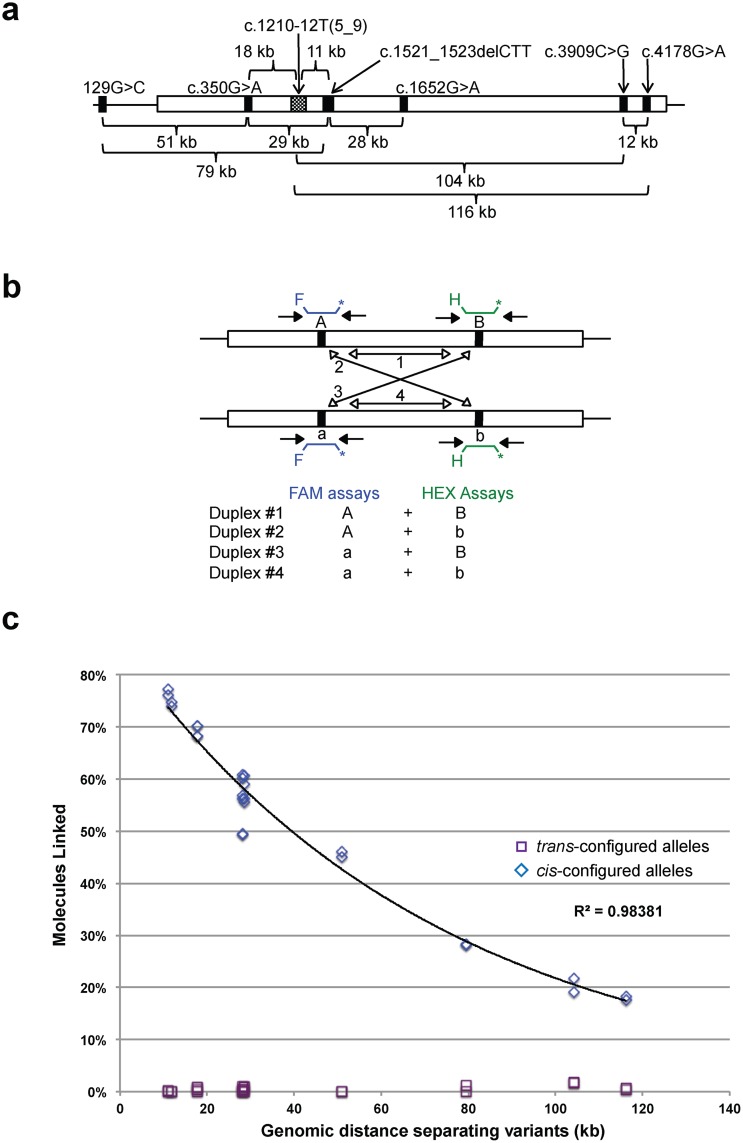
Phasing *CFTR* variants in the genomes of cystic fibrosis patients. (**a**) Locations and genomic distances separating the variants along the *CFTR* gene in the tested cell lines. (**b**) Assembly of four duplex assays to redundantly evaluate phase of screened variants. (**c**) Physical linkage of *CFTR* variants as measured by Drop-Phase, as a function of genomic distance (horizontal axis). Blue diamonds: allele-pairs inferred to be *cis*-configured; purple squares: allele-pairs inferred to be *trans*-configured. The black line is an exponential curve fit to the *cis*-configured allele-pairs. Four duplex assays were performed per variant pair. Variants were classified as *cis*- or *trans*-configured based on measured positive linkage or lack of linkage, respectively. Samples were analyzed in duplicate.

We analyzed genomic DNA derived from cell lines from six cystic fibrosis patients; each of these patients was previously known to be heterozygous for two to four *CFTR* variants (nine variants total, across the six patients, because several variants were shared by multiple patients) (Methods). The identities and genomic locations of these variants are shown in Fig. 3a. The genomic distance separating pairs of variants heterozygous in the same individual ranged from 12 to 116 kb.

For each of the six variant pairs tested, we tested for all four possible allelic configurations. More specifically, in an *Aa*:*Bb* compound heterozygote, we tested for the haplotypes *A-B*, *A-b*, *a-B*, and *a-b*. Note that this involves some redundancy (because the existence of an *A-B* haplotype implies the existence of an *a-b* haplotype on the other chromosome) (Fig. 3b). Such redundant assays offered opportunities to critically evaluate Drop-Phase, since the inference (for example) of an *A-B* and an *A-b* haplotype in an *Aa*:*Bb* compound heterozygote must be incorrect.

For all (13/13) variant pairs heterozygous in any of the six individuals, we were able to infer both haplotypes (Table 1). Within each individual, results were in every case internally consistent in the sense that the inferred haplotypes contained opposite alleles (Table 1). In addition, we found that these haplotype inferences were in each case consistent with these individuals’ known status as cystic fibrosis patients. Three of the six patients (GM11286, GM11274, and GM03465) had recessive loss-of-function variants in the *trans* configuration (ΔF508 on one chromosome; c.1652G>A on the other chromosome). Two other patients (GM13591 and GM11279) had ΔF508 *trans*-configured to a complex haplotype of multiple milder variants (*cis*-configured [350G>A; 1210–12T(5)]), which in one of these individuals (GM11279) was also *cis*-configured to 129G>C. The sixth individual (GM11472) had two multi-variant haplotypes ([c.3909C>G; 1210–12T(9)] on one chromosome, and [c.4046G>A; 1210–12T(7)] on the other chromosome).

**Table 1:** Haplotypes formed by *CFTR* variants in six cystic fibrosis patients.

Variant	129G/C	R117H	5T	7T	9T	ΔF508	G551D	N1303K	G1349D
Effect on cDNA	promoter	350G>A	intron	intron	intron	1521_1523 delCTT	1652G>A	3909C>G	4046G>A (4178G>A)
GM11286						Hap 1	Hap 2		
GM03465						Hap 1	Hap 2		
GM11274						Hap 1	Hap 2		
GM11279	Hap 1	Hap 1	Hap 1		Hap 2	Hap 2			
GM11472				Hap 1	Hap 2			Hap 2	Hap 1
GM13591		Hap 1	Hap 1		Hap 2	Hap 2			

Key: Hap 1 = Haplotype 1, Hap 2 = Haplotype 2

The varied genomic spacing among the variant pairs (11–116 kb) made it possible to analyze how measurements of physical linkage related to genomic distance (Fig. 3c). Assay pairs for the two closest variants (c.1521_1523 and c.1210–12T(5_9), separated by 11 kb), gave the greatest percentage of linked copies (77%), whereas assay pairs for the two most distant variants (c.1210–12T(5_9) and c.4046, separated by 116 kb) gave the smallest percentage of linked copies (18%), although this was still far greater than the largest percentage in any unlinked case (< 2%). Moreover, across all pairs of assays, measurements of physical linkage closely followed the expected exponential relationship between genomic distance and physical linkage (Fig. 3c).

### Robustness of Drop-Phase to SNP assay designs

We sought to make Drop-Phase easy, scalable, and functional for almost any pair of sequence variants. A potential challenge in discriminating SNPs arises when a fluorescence reporter (such as allele-specific hydrolyzable probes designed to a “targeted” allele) cross-reacts with the non-targeted allele at an appreciable rate. In many such cases, such cross-reaction resulted in the presence of additional clusters of droplets with intermediate levels of fluorescence. Although specificity can be achieved with careful assay design, such as the use of “dark” (non-fluorescent) competitor probes to reduce cross-reactivity, we sought to enhance the robustness of Drop-Phase to cases in which the assays are somewhat responsive to a non-targeted allele.

**Fig 4:**
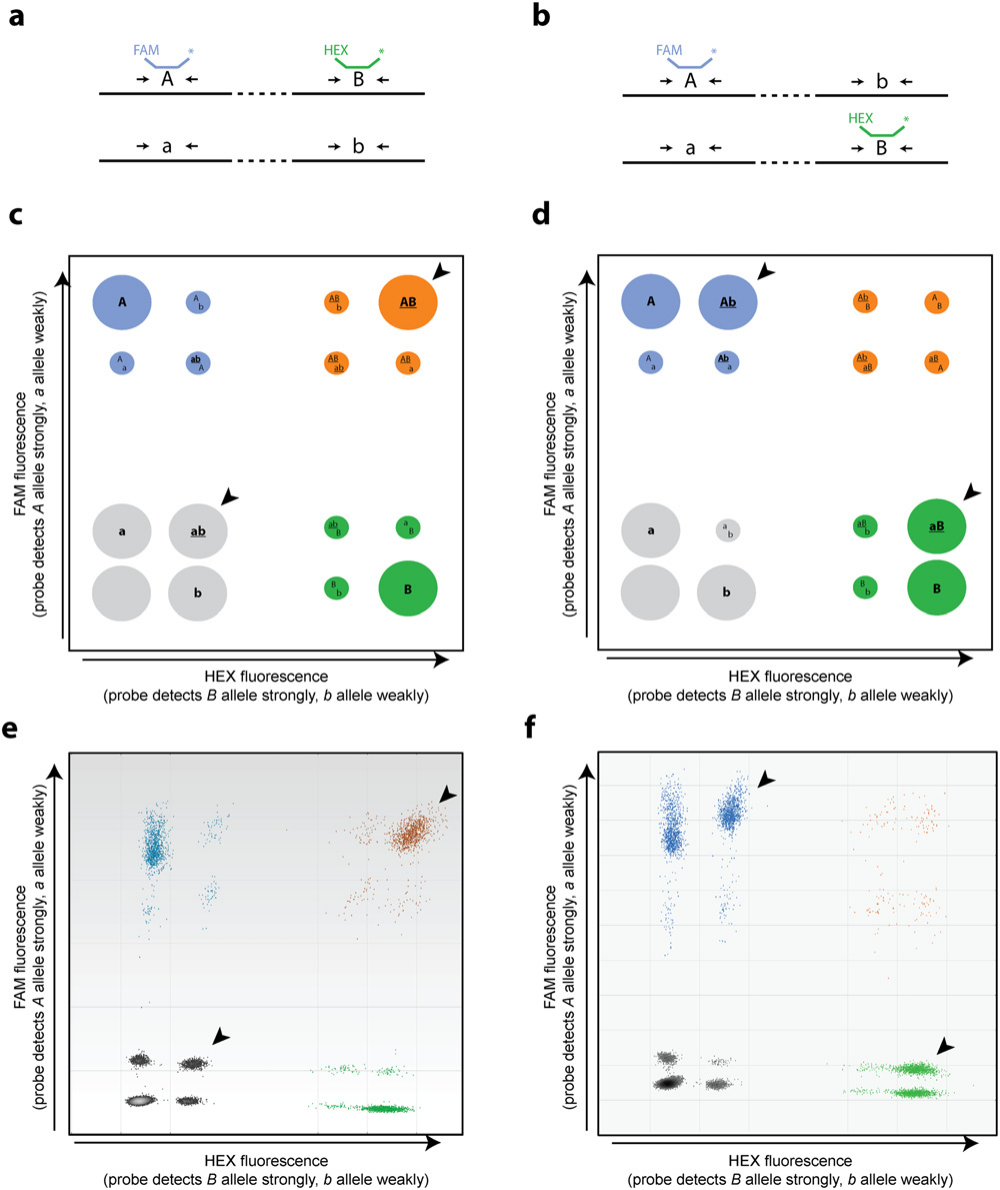
Droplet cluster identification and classification in the context of allelically cross-reacting fluorescence probes. **(a,b)** The two potential haplotype configurations in a compound heterozygote. Primer pairs (arrows) are designed for both loci, and fluorescent probes are designed for the *A* allele at locus *A/a* and the *B* allele at locus *B/b*. **(c,d)** Expected populations of droplets under the two potential haplotype configurations in panels **a** and **b**. Although fluorescence probes are designed to one allele, they also fluoresce (at reduced intensity) in response to the other allele. For example, when a FAM-labeled probe is designed to the *A* allele, droplets exhibit four levels of FAM fluorescence: the highest level for droplets containing only the *A* allele; a lower level for droplets containing a mixture of *A* and *a*; a substantially lower level for droplets containing only *a*; and the lowest level for droplets containing neither *A* nor *a* (S3 Fig.). When both SNP assays have this property, up to 16 (2^4^) different patterns of fluorescence will be detected, depending on the presence or absence in each droplet of targeted (*A*, *B*) alleles and non-targeted (*a*, *b*) alleles. Droplets arising from a single molecular species (e.g., the linked *AB* species) are more common than droplets arising from combinations of molecules that happen by chance to appear in the same droplet (e.g., unlinked molecules containing *A* and *b*). Arrowheads indicate common droplet populations that are diagnostic of the key linked species (*AB* and *ab* in the first individual; *Ab* and *aB* in the second). **(e,f)** Drop-Phase data diagnostic of the two different haplotypic configurations in panels **a** and **b**. Arrowheads indicate the highly populated clusters diagnostic of the linked species. Mathematical analysis of the droplet population sizes (S1 Note) is used to estimate the number of linked molecules of each species and determine phase. S4 Fig. elaborates on the relationship of these droplet population sizes to DNA input concentration.

Assays that fluoresce in response to both targeted and non-targeted alleles result in more complex populations of droplets; when both SNP assays have this property, up to 16 (2^4^) different patterns of fluorescence will be detected, depending on the presence or absence in each droplet of targeted (*A*, *B*) alleles and non-targeted (*a*, *b*) alleles. These patterns are readily distinguished on a droplet-intensity scatter plot (Fig. 4, S3 Fig., S4 Fig.). For example, consider a heterozygous site (*A/a*) analyzed using a FAM-labeled probe that targets the *A* allele but also responds to the *a* allele at a lower intensity (due to a lower rate of probe hybridization and hydrolysis). Droplets containing only amplicons with the *A* allele exhibit the highest level of FAM fluorescence (Fig. 4c-f, S3 Fig., S4 Fig.; note the two blue and two orange clusters across the top), whereas droplets containing a mixture of amplicons with the two alleles (*A* and *a*) exhibit a lower level of FAM fluorescence (Fig. 4c-f, S3 Fig., S4 Fig.). Droplets containing only the non-targeted *a* allele exhibit a much lower level of fluorescence, and droplets containing neither allele have the lowest FAM fluorescence (Fig. 4c, d, S3 Fig., S4 Fig.; note the clusters shown in gray and green). An equivalent set of relationships characterizes the other fluorophore (HEX) and the other locus (*B/b*) (Fig. 4, S3 Fig., S4 Fig.).

To phase sequence variants, it is necessary only to distinguish those droplets that are positive for the targeted allele from those that lack the targeted allele, i.e., to distinguish *A*-only and *A+a* droplets from *a*-only and *0* droplets, and *B*-only and *B+b* droplets from *b*-only and *0* droplets. This is readily accomplished by treating the 16 droplet populations (clusters) as four meta-populations (meta-clusters; shown in blue, green, orange, and gray in Fig. 4); conveniently, these four meta-populations correspond to the ways in which the droplet intensities already cluster in two-dimensional fluorescence space (Fig. 4). Surprisingly, we found that such cross-reacting assays actually provided additional information, because in such cases a single duplex assay could identify all four linked species (*AB*, *Ab*, *aB*, and *ab*) (Fig. 4c-f).

## Conclusions

We developed a method, Drop-Phase, for quickly evaluating the chromosomal phase of pairs of DNA sequence variants by massively partitioning individual reaction vessels (wells) into droplets and evaluating the co-partitioning of sequences into droplets. Drop-Phase is rapid (requiring less than 4 hours), scalable (1–96 samples), and effective at substantial genomic distances (200 kb). Drop-Phase is also technically easy to perform and low in cost (S3 Table).

The genomic distance at which variants can be phased by Drop-Phase (200 kb, even in conventionally extracted DNA samples) exceeds the lengths of 94% of human protein-coding genes. Analyzing a series of pairs of genomic variants, with transitive inference of chromosomal phase, would allow phasing at still-larger scales, limited primarily by DNA quality and the spacing of heterozygous sites in an individual’s genome. We believe that Drop-Phase could thereby be used to phase variants of interest in almost any human gene.

Drop-Phase also has important limitations. Though it scales quickly to large numbers of samples, it does not scale quickly to large numbers of loci, as each assay requires its own allele-specific fluorescence probes and optimization. The primary application of Drop-Phase will therefore be in phasing specific variants of interest to a researcher or clinical geneticist. These variants will generally first be ascertained by other methods, such as whole-exome or whole-genome sequencing, gene-specific sequencing, or genome-wide genotyping. The genomic range at which Drop-Phase can phase variants is limited by sample preparation; multi-well analysis did not substantially extend this range beyond the distances reported here (data not shown), and we believe that DNA extraction methods are the most promising way to extend genomic range for applications for which longer-range phasing is important.

Drop-Phase will benefit allele-specific expression studies, identification of complex alleles and compound heterozygotes, mapping of hard-to-resolve genome structures, characterization of genome edits and *de novo* mutations, and many other applications.

## Supporting Information

S1 Fig Molecular species contributing to droplets of each type, under linked and unlinked scenarios. (PDF)

S2 Fig Physical intactness of DNA extracted using silica columns. (PDF)

S3 Fig Complex populations of droplet-clusters arising when allele-specific assays cross-react with non-targeted alleles. (PDF)

S4 Fig Effect of DNA input concentration on droplet populations. (PDF)

S1 Note Mathematical relationships underlying linkage and droplet populations. (PDF)

S1 Table Assays used to assess physical linkage as a function of genomic distance. (PDF)

S2 Table Assays used to analyze *CFTR*. (PDF)

S3 Table Laboratory costs associated with Drop-Phase. (PDF)
