# Supplementary material for: A Rapid Molecular Approach for Chromosomal Phasing
Source: PLoS One. 2015 Mar 4;10(3):e0118270. doi: 10.1371/journal.pone.0118270 (PMC4349636; doi:10.1371/journal.pone.0118270)
Supplement: S1 Fig — (PDF) [file pone.0118270.s001.pdf]

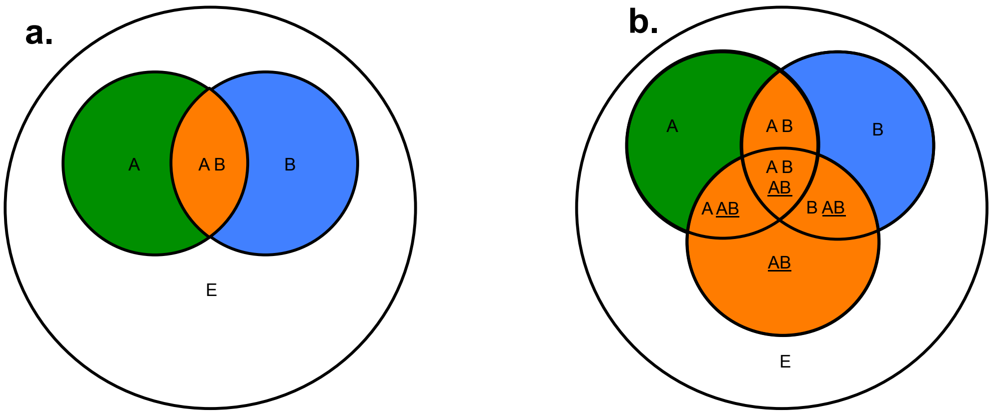

Figure S1

These Venn diagrams show the kinds of droplet species expected when the two detected molecules (*A* and *B*) are unlinked (panel **a**) or linked (panel **b**). Green indicates *A*-only droplets. Blue indicates *B*-only droplets. Orange indicates droplets containing both *A* and *B*.

(**a**) When the molecular species are unlinked, there are four categories of droplets: *A, B, A*+*B* and *E* (empty) droplets. Double-positive droplets (orange) arise only when *A* and *B* co-partition into the same droplet by chance.

(**b**) When the molecular species are linked, there are additional categories of double-positive droplets containing the linked species <u>*AB*</u> – on its own, or in combination with the unlinked *A* and *B* species that exist due to DNA fragmentation. Double positives can arise from any of these combinations (*A*+*B*), (*A*+<u>*AB*</u>), (*B*+<u>*AB*</u>), (*A*+*B*+<u>*AB*</u>), and (<u>*AB*</u>). Note that we directly observe only whether a droplet is positive or negative for *A* or *B* – the various classes of droplets shown in orange are not distinguishable in the assay. The mathematical analysis in **Supplementary Note** is therefore necessary to estimate the concentrations of the linked and unlinked species.
