# Supplementary material for: A Rapid Molecular Approach for Chromosomal Phasing
Source: PLoS One. 2015 Mar 4;10(3):e0118270. doi: 10.1371/journal.pone.0118270 (PMC4349636; doi:10.1371/journal.pone.0118270)
Supplement: S2 Fig — (PDF) [file pone.0118270.s002.pdf]

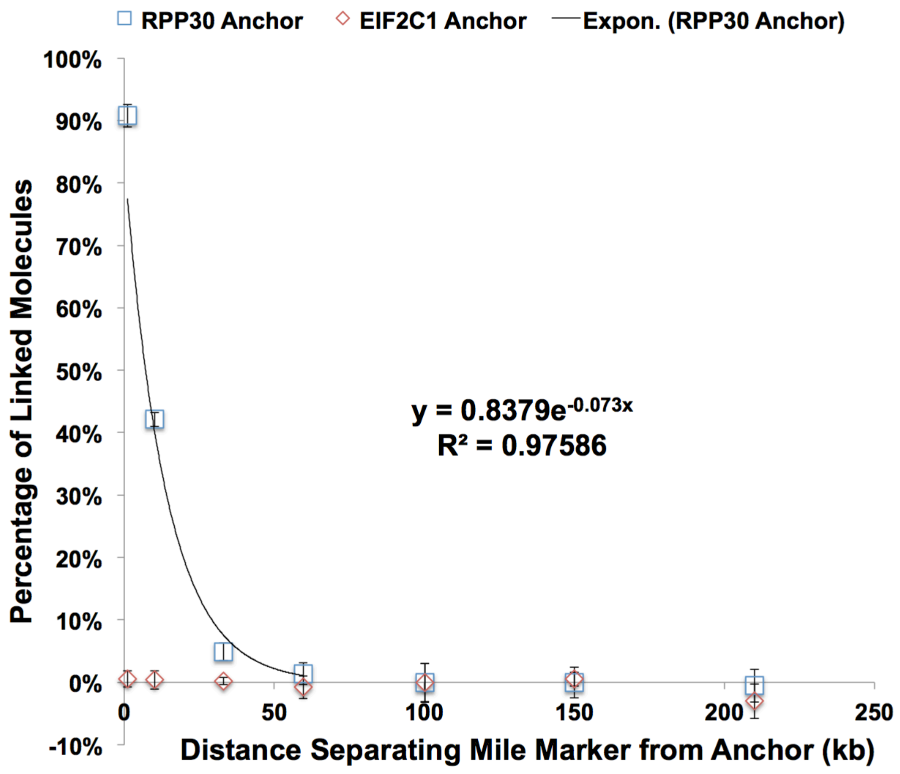

Figure S2. Physical intactness of DNA extracted using silica columns. (Intactness for DNA extracted using a polysaccharide precipitation kit is reported in **Fig. 2**). From triplicate ddPCR measurements, the mean (and 95% confidence interval) of percent molecules linked for 1, 10, 33, 60, 100, 150, and 210 kb distances are shown as a function of genomic distance. (**Fig. 2** reported the intactness of DNA samples isolated using the polysaccharide precipitation method.)
