# Supplementary material for: A Rapid Molecular Approach for Chromosomal Phasing
Source: PLoS One. 2015 Mar 4;10(3):e0118270. doi: 10.1371/journal.pone.0118270 (PMC4349636; doi:10.1371/journal.pone.0118270)

Figure S3. Complex populations of droplet-clusters arising when allele-specific assays cross-react with non-targeted alleles.

(**a**) Although fluorescence probes are designed to one allele, they may also fluoresce (at reduced intensity) in response to the other allele. This gives rise to complex populations of as many as 16 droplet clusters.

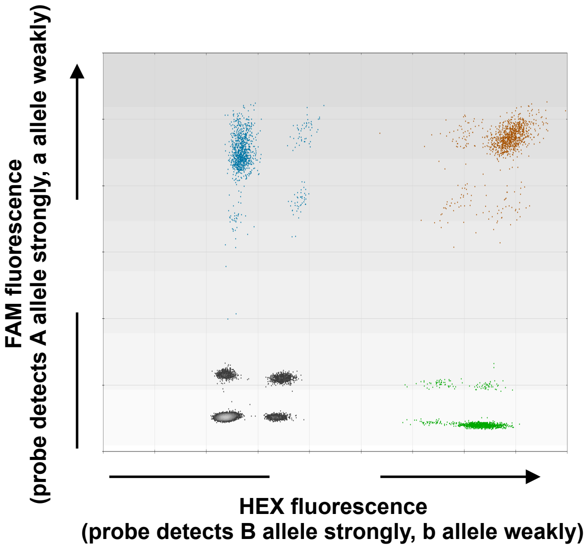

(**b**) When a FAM-labeled probe designed to the *A* allele shows some (albeit weak) response to the *a* allele, droplets exhibit four levels of fluorescence: the highest level for droplets containing only the *A* allele; a lower level for droplets containing a mixture of *A* and *a*; a substantially lower level for droplets containing only *a*; and the lowest level for droplets containing neither *A* nor *a*.

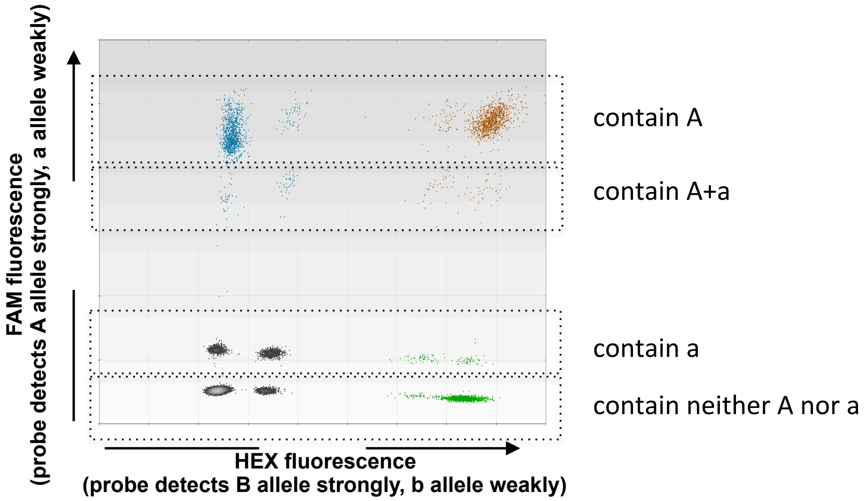

(**c**) When a HEX-labeled probe designed to the *B* allele shows some (albeit weak) response to the *b* allele, droplets exhibit four levels of fluorescence: the highest level for droplets containing only the *B* allele; a lower level for droplets containing a mixture of *B* and *b*; a substantially lower level for droplets containing only *b*; and the lowest level for droplets containing neither *B* nor *b*.

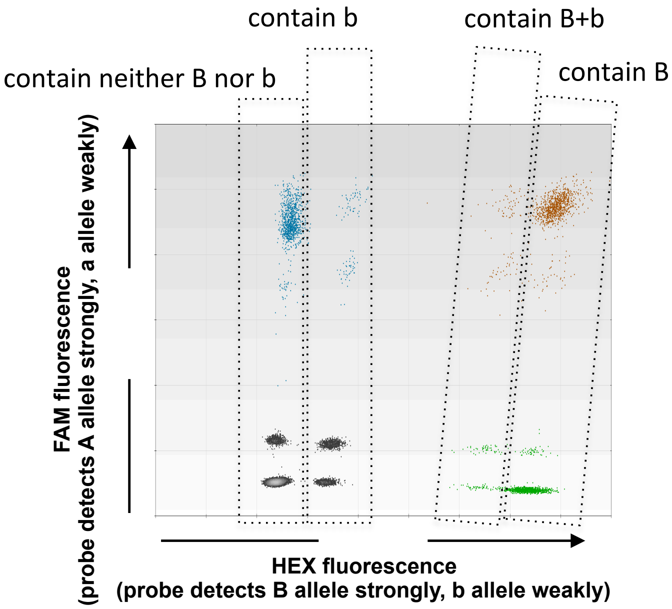

Supplement: S3 Fig — (PDF) [file pone.0118270.s003.pdf]
