# Supplementary material for: A Rapid Molecular Approach for Chromosomal Phasing
Source: PLoS One. 2015 Mar 4;10(3):e0118270. doi: 10.1371/journal.pone.0118270 (PMC4349636; doi:10.1371/journal.pone.0118270)

Figure S4. Effect of DNA input concentration on droplet populations.
We assessed the performance of Drop-Phase across a range of DNA inputs.

(**a,b**) The two potential haplotype configurations in a compound heterozygote.

a

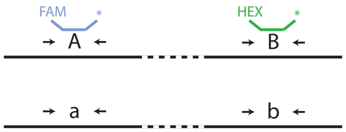

b

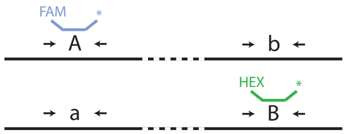

(**c,d**) Schematic showing the major droplet populations expected for *cis*- configured alleles (**c**) and *trans*-configured alleles (**d**) at low DNA input levels. At these DNA input levels, each droplet tends to contain a single molecular species; arrowheads denote the key linked species that are diagnostic of chromosomal phase.

c

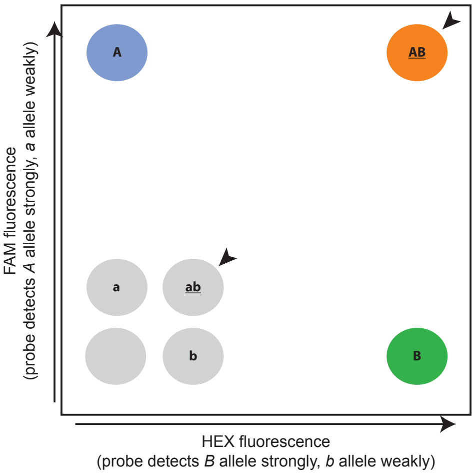

d

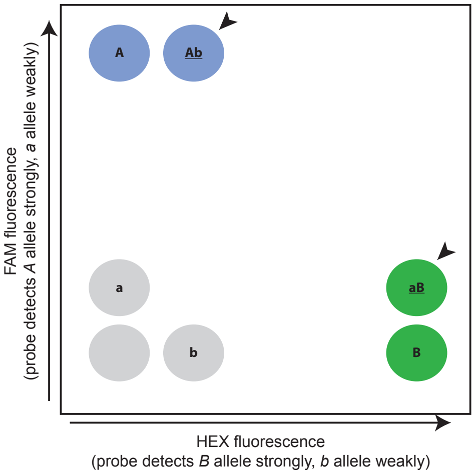

(**e,f**) Droplet 2D plots for *cis*- and *trans*-configured alleles (respectively) at low DNA input levels. At these low DNA input levels, just seven clusters are observed, and chromosomal phase is apparent from the presence of the key linked species (arrowheads)

e

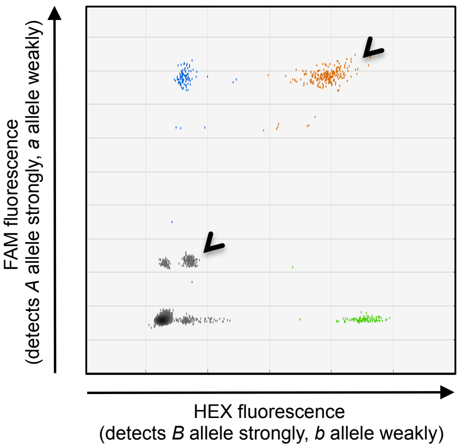

f

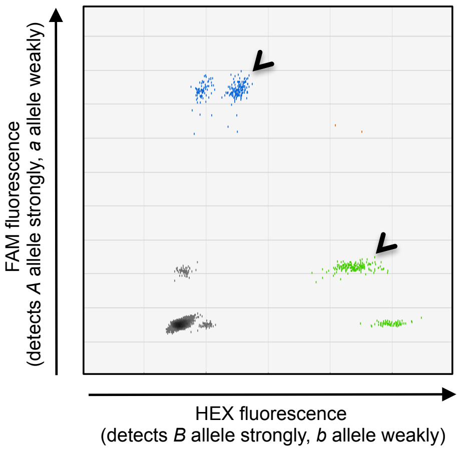

(**g,h**) Schematic showing the major droplet populations expected for *cis*- configured alleles (**c**) and *trans*-configured alleles (**d**) at higher DNA input levels. Note that droplets containing combinations of molecular species (rather than individual molecular species) become much more common when DNA input is high.

g **h**

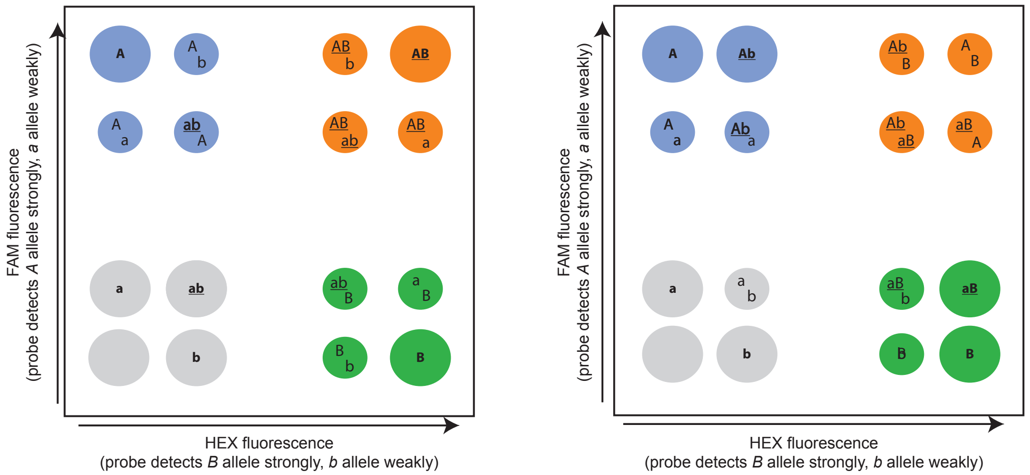

(**i,j**) Droplet 2D plots for cis-configured (**i**) and trans-configured (**j**) alleles at high DNA input levels. At these higher genomic DNA input levels, additional clusters (arising from combinations of molecular species) became apparent due to chance co-localization. Although higher DNA input levels made it more difficult to visually distinguish the two potential haplotype configurations, mathematical analysis (**Supplementary Note**) of the numbers of droplets in the four droplet meta-populations (shown in gray, blue, green, and orange, and corresponding respectively to droplets that are *A-B-, A+B-, A-B+*, and *A+B+*; see **Supplementary Fig. 3**) reached the same conclusion about linkage at all DNA input levels. Across a four-point, four-fold dilution series from 1536 to 24 copies/µL, estimates of the concentration of linked molecules (**Supplementary Note**) varied by less than 4%, despite substantial differences in the numbers of droplets in each cluster.

i **j**

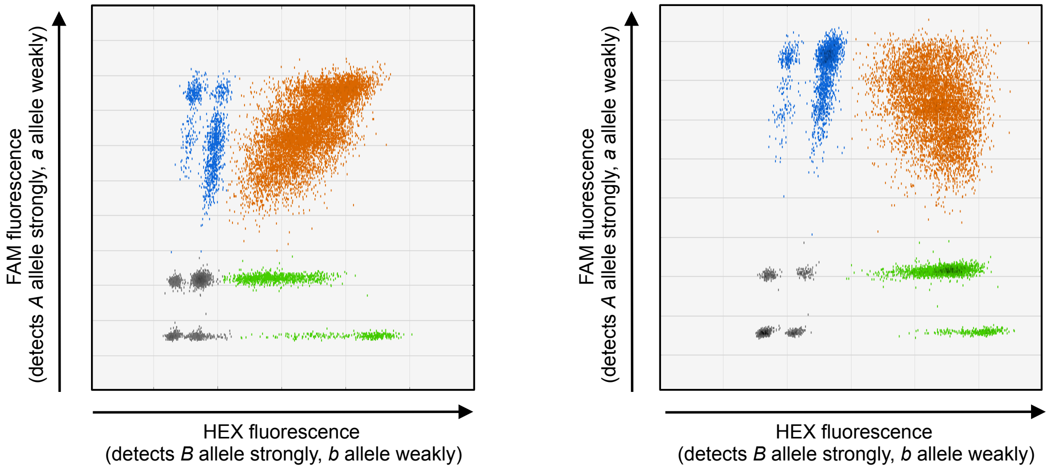

Supplement: S4 Fig — (PDF) [file pone.0118270.s004.pdf]
