# Supplementary material for: A Rapid Molecular Approach for Chromosomal Phasing
Source: PLoS One. 2015 Mar 4;10(3):e0118270. doi: 10.1371/journal.pone.0118270 (PMC4349636; doi:10.1371/journal.pone.0118270)
Supplement: S1 Note — (PDF) [file pone.0118270.s005.pdf]

Note S1: Mathematical relationships underlying linkage and droplet populations

All DNA fragments are distributed into the droplets independent of one another. In the case where the two markers (*A*) and (*B*) are not physically on the same chromosome (or are always unlinked), we still expect to see partitions that will have both (*A*) and (*B*) present purely due to chance co-localization. The independence of molecular partitioning is shown in **Supplementary Fig. 1a** and expressed in:

Eq. 1 $N_A N_B = N_E N_{AB}$

where N denotes the number of droplets, so that N_A and N_B are the counts of single-positive droplets, N_E is the count of negative (empty) droplets, and N_{AB} is the count of double-positive droplets. Some droplets will contain multiple copies of the same target.

In experiments where no linkage is anticipated, we expect **Eq. 1** to be true on average due to the random distribution of targets into droplets.

If we want to estimate the number of droplets observed as double-positives due to chance, N_{ch}, we can rewrite **Eq. 1** as:

Eq. 2 $N_{ch} = N_A N_B / N_E$

We now consider the case where the two DNA sequences are physically linked. When linked $\underline{AB}$ molecules are present (an underlined $\underline{AB}$ denotes a linked molecule) there will be extra double-positive droplets. **Supplementary Fig. 1b** shows the distribution of all possible combinations of targets into droplets. Note that the double positive droplets can come in 5 different types: (A+B), (A+$\underline{AB}$), (B+$\underline{AB}$), ($\underline{AB}$) and (A+B+$\underline{AB}$). The total number of droplets that do not contain linked molecules is calculated using:

Eq. 3 $N_{not\,\underline{AB}} = N_E + N_A + N_B + N_A N_B / N_E$

From this we can calculate the concentration (average copies/droplet) of the molecules contributing to the linked droplets ($\underline{AB}$) using:

Eq. 4 $\lambda_{\underline{AB}} = ln(N_{tot}) - ln(N_E + N_A + N_B + N_A N_B / N_E)$

Where N_{tot} is the total number of observed droplets. When $\lambda_{\underline{AB}}$ is close to zero, it may evaluate to a negative number due to stochastic effects.

We calculate the average copies of each molecular species (A and B, irrespective of linkage) per droplet, using:

Eq. 5a $\lambda_A = ln(N_{tot}) - ln(N_{not\,A})$
Eq. 5b $\lambda_B = ln(N_{tot}) - ln(N_{not\,B})$

Where $N_{not\,A}$ and $N_{not\,B}$ is the total number of droplets not containing A or not containing

B, respectively. The concentration estimates for *A* and *B* may be slightly different due to molecular sampling, and occasionally due also to differences in target accessibility, amplicon size, or fine DNA fragmentation structure.

To compute the percent linked molecules (*%linkage*), we divide the estimated linked concentration by the average measured concentration of *A* and *B*, and convert to percentage:

Eq. 6 $\%\underline{AB} = (2\lambda_{\underline{AB}}/(\lambda_A+\lambda_B))100$
