# Supplementary material for: A Rapid Molecular Approach for Chromosomal Phasing
Source: PLoS One. 2015 Mar 4;10(3):e0118270. doi: 10.1371/journal.pone.0118270 (PMC4349636; doi:10.1371/journal.pone.0118270)
Supplement: S1 Table — (PDF) [file pone.0118270.s006.pdf]

Table S1. “Mile marker” assays used to assess physical linkage as a function of genomic distance

Mile marker assays	Forward primer	Reverse primer	Probe
RPP30_6 (Anchor)	GATTTGGACCTGCGAGCG	GCGGCTGTCTCCACAAGT	HEX-TCTGACCTGAAGGCTCTGCGCG-IABkFQ
MM_1K	GGCGCTAATGTTCTTTGATCCTGTA	CGTCAGGAAAAGCCTCATTGAG	6-FAM-ATTGCGACAAACTACA-MGB
MM_10K	CTCCCTCTCCATAGCTACTTAAGGA	CAAGGAGCCCTAACCAATGGA	6-FAM-AAGGCAGAGATTAAAG-MGB
MM_33K	GCCGAGCATGGTCATCAAAG	GTACTTTCCTACTTGTTGTTATGTAGAGACAA	6-FAM-CCAGAAAACAAAGTTTGAT-MGB
MM_60K	GGAGAGTGCTTTCCCATGTT	TAACCTCCACACTCACTGGT	6-FAM-CCCCTCAGTCACTCTGGCAT-IABkFQ
MM_100K	CACCGGAGTTGGACATGTGATAAT	GACTTTTCCATCTCAGTAGGAACAGAA	6-FAM-CACACGGAAGTATATG-MGB
MM_150K	CTCAGCATCACTGTGCCATA	ACGACGGAGGAATACAGCTA	6-FAM-TCAGGCCTCCAAAATCTGCCT-IABkFQ
MM_210K	TTCAGAAGCCCACAACTCTG	TACCTCATGTCCTTACCCCT	6-FAM-AGTCTCTGTTCTGTCTTTGGCCA-IABkFQ

All sequences listed 5’-3’. For the mile marker experiments, the negative control anchor assay (EIF2C1, unique assay ID: dHsaCP1000002, Bio-Rad), targets an ultra-conserved element in the EIF2C1 gene at chromosome location 1: 36359351-36359473.
