# Supplementary material for: A Rapid Molecular Approach for Chromosomal Phasing
Source: PLoS One. 2015 Mar 4;10(3):e0118270. doi: 10.1371/journal.pone.0118270 (PMC4349636; doi:10.1371/journal.pone.0118270)
Supplement: S2 Table — (PDF) [file pone.0118270.s007.pdf]

Table S2. *CFTR* assays

***CFTR* phasing assays**	**Forward primer**	**Reverse primer**	**Fluorescent probe**	**Competitor probe**
CFTR_129G<br>wt.FAM	CAGACGG<br>CCCTAGCA	CCTTTTCC<br>AGAGGCG<br>AC	wt.FAM 6-FAM-<br>CTGCATGGTcTCTCGG-<br>IABkFQ	mut.dark<br>CATGGTgTCTCGGGC-<br>IABkFQ
CFTR_129G>C<br>mut.FAM	CAGACGG<br>CCCTAGCA	CCTTTTCC<br>AGAGGCG<br>AC	mut.FAM 6-FAM-<br>CATGGTgTCTCGGGC-<br>IABkFQ	wt.dark<br>CTGCATGGTcTCTCGG-<br>IABkFQ
CFTR_c.350G<br>(R117)<br>wt.FAM	CTTCCTAT<br>GACCCGG<br>ATAAC	CATAAGC<br>CTATGCCT<br>AGATAAA	wt.FAM<br>6-FAM-<br>AGGAACgCTCTATCGC-<br>IABkFQ	mut.dark<br>AGGAACaCTCTATCGCG-<br>IABkFQ
CFTR_c.350G>A<br>(R117H)<br>mut.FAM	CTTCCTAT<br>GACCCGG<br>ATAAC	CATAAGC<br>CTATGCCT<br>AGATAAA	mut.FAM 6-FAM-<br>AGGAACaCTCTATCGCG<br>-IABkFQ	wt.dark<br>AGGAACgCTCTATCGC-<br>IABkFQ/
CFTR_c.350G<br>(R117)<br>wt.HEX<br>sku3	CTTCCTAT<br>GACCCGG<br>ATAAC	CATAAGC<br>CTATGCCT<br>AGATAAA	wt.HEX HEX-<br>AGGAACgCTCTATCGC-<br>IABkFQ	mut.dark<br>AGGAACaCTCTATCGCG-<br>IABkFQ
CFTR_c.350G>A<br>(R117H)<br>mut.HEX	CTTCCTAT<br>GACCCGG<br>ATAAC	CATAAGC<br>CTATGCCT<br>AGATAAA	mut.HEX HEX-<br>AGGAACaCTCTATCGCG<br>-IABkFQ	wt.dark<br>AGGAACgCTCTATCGC-<br>IABkFQ
CFTR_7T<br>wt.HEX when other allele is 5T	ATCTATTG<br>AAAATATC<br>TGACAAA<br>CT	CTTTCTCA<br>AATAATTC<br>CCCAAATC	wt.HEX HEX-<br>TGTGTGTGtttttttAACAGG<br>-IABkFQ	5T_mut.dark<br>TGTGTGTGTGtttttAACAG-<br>IABkFQ
CFTR_7T<br>wt.HEX when other allele is 9T	AGCATCTA<br>TTGAAAAT<br>ATCTGAC	CTCAAATA<br>ATTCCCCA<br>AATCC	wt.HEX HEX-<br>TGTGTGTGtttttttAACAGG<br>-IABkFQ	9T_mut.dark<br>TGTGTGtttttttttAACAGGG-<br>IABkFQ
CFTR_5T<br>mut.HEX when other allele is<br>7T	ATCTATTG<br>AAAATATC<br>TGACAAA<br>CT	CTTTCTCA<br>AATAATTC<br>CCCAAATC	mut.HEX HEX-<br>TGTGTGTGTGtttttAACAG<br>-IABkFQ	7T.wt.dark<br>TGTGTGTGtttttttAACAGG-<br>IABkFQ
CFTR_9T<br>mut.HEX when other allele is<br>7T	AGCATCTA<br>TTGAAAAT<br>ATCTGAC	CTCAAATA<br>ATTCCCCA<br>AATCC	mut.HEX HEX-<br>TGTGTGtttttttttAACAGGG<br>-IABkFQ	7T.wt.dark<br>TGTGTGTGtttttttAACAGG-<br>IABkFQ
CFTR_c.1521_1523<br>(F508)<br>wt.FAM	ATTATGCC<br>TGGCACC<br>ATTA	TGATGACG<br>CTTCTGTA<br>TCTA	wt.FAM 6-FAM-<br>AATATCATcttTGGTGTTT<br>CCT-IABkFQ	mut.dark<br>AATATCATTGGTGTTTCC<br>TATGA-IABkFQ
CFTR_c.1521_1523del3<br>(F508del)<br>mut.FAM	ATTATGCC<br>TGGCACC<br>ATTA	TGATGACG<br>CTTCTGTA<br>TCTA	mut.FAM 6-FAM-<br>AATATCATTGGTGTTTC<br>CTATGA-IABkFQ	wt.dark<br>AATATCATcttTGGTGTTT<br>CCT-IABkFQ

CFTR_c.1521_1523<br>(F508)<br>wt.HEX	ATTATGCC<br>TGGCACC<br>ATTA	TGATGACG<br>CTTCTGTA<br>TCTA	wt.HEX HEX-<br>AATATCATcttTGGTGTTT<br>CCT-IABkFQ	mut.dark<br>AATATCATTGGTGTTTCC<br>TATGA-IABkFQ
CFTR_c.1521_1523del3<br>(F508del)<br>mut.HEX	ATTATGCC<br>TGGCACC<br>ATTA	TGATGACG<br>CTTCTGTA<br>TCTA	mut.HEX HEX-<br>AATATCATTGGTGTTTC<br>CTATGA-IABkFQ	wt.dark<br>AATATCATcttTGGTGTTT<br>CCT-IABkFQ
CFTR_c.1652G<br>(G551)<br>wt.FAM	GGAGAAG<br>GTGGAATC<br>ACA	TTATTCAC<br>CTTGCTAA<br>AGAAAT	wt.FAM 6-FAM-<br>TGAGTGGAGgTCAACGA<br>-IABkFQ	mut.dark<br>AGTGGAGaTCAACGAGC-<br>IABkFQ
CFTR_c.1652G>A<br>(G551D)<br>mut.FAM	GGAGAAG<br>GTGGAATC<br>ACA	TTATTCAC<br>CTTGCTAA<br>AGAAAT	mut.FAM 6-FAM-<br>AGTGGAGaTCAACGAGC<br>-IABkFQ	wt.dark<br>TGAGTGGAGgTCAACGA-<br>IABkFQ
CFTR_c.3909C<br>(N1303)<br>wt.FAM	AGGGAAA<br>AATAAAA<br>AGTTATTT<br>AAG	TCCATATT<br>TCTTGATC<br>ACTCC	wt.FAM 6-FAM-<br>AAAAAAcTTGGATCCCT<br>ATGAA-IABkFQ	mut.dark<br>AAAAAAgTTGGATCCCTA<br>TGAA-IABkFQ
CFTR_c.3909C>G<br>(N1303K)<br>mut.FAM	AGGGAAA<br>AATAAAA<br>AGTTATTT<br>AAG	TCCATATT<br>TCTTGATC<br>ACTCC	mut.FAM 6-FAM-<br>AAAAAAgTTGGATCCCT<br>ATGAA-IABkFQ	wt.dark<br>AAAAAAcTTGGATCCCTA<br>TGAA-IABkFQ
CFTR_c.4046G<br>(c.4046G is also referred to as c.4178G in some dbSNP databases)<br>(G1349)<br>wt.HEX	GACTTTGT<br>CCTTGTGG<br>ATG	ATCTAGCC<br>AAGCACA<br>TCA	wt.HEX HEX-<br>AAGCCATGgCCACAAG-<br>IABkFQ	mut.dark<br>AGCCATGaCCACAAGC-<br>IABkFQ
CFTR_c.4046G>A<br>(c.4046G>A is also referred to as c.4178G>A in some dbSNP databases)<br>(G1349D)<br>mut.HEX	GACTTTGT<br>CCTTGTGG<br>ATG	ATCTAGCC<br>AAGCACA<br>TCA	mut.HEX HEX-<br>AGCCATGaCCACAAGC-<br>IABkFQ	wt.dark<br>AGCCATGgCCACAAG-<br>IABkFQ
CFTR_c.4046G<br>(c.4046G is also referred to as c.4178G in some dbSNP databases)<br>(G1349)<br>wt.FAM	GACTTTGT<br>CCTTGTGG<br>ATG	ATCTAGCC<br>AAGCACA<br>TCA	wt.FAM 6-FAM-<br>AAGCCATGgCCACAAG-<br>IABkFQ	mut.dark<br>AGCCATGaCCACAAGC-<br>IABkFQ
CFTR_c.4046G>A<br>(c.4046G>A is also referred to as c.4178G>A in some dbSNP databases)<br>(G1349D)<br>mut.FAM	GACTTTGT<br>CCTTGTGG<br>ATG	ATCTAGCC<br>AAGCACA<br>TCA	mut.FAM 6-FAM-<br>AGCCATGaCCACAAGC-<br>IABkFQ	wt.dark<br>AGCCATGgCCACAAG-<br>IABkFQ

All sequences listed 5’-3’.
