# Supplementary material for: A Rapid Molecular Approach for Chromosomal Phasing
Source: PLoS One. 2015 Mar 4;10(3):e0118270. doi: 10.1371/journal.pone.0118270 (PMC4349636; doi:10.1371/journal.pone.0118270)
Supplement: S3 Table — (PDF) [file pone.0118270.s008.pdf]

Table S3: Laboratory costs associated with Drop-Phase

Below are costs associated with our implementation of Drop-Phase; we note that reagents and systems for digital PCR are also available from other vendors. Different cost components scale with the numbers of samples and SNPs analyzed. Costs may scale best in scenarios in which a laboratory has genes of long-term interest and analyzes the chromosomal phase of the same variants in many different samples.

Per sample analyzed	Digital PCR consumables (e.g. emulsion cartridge, gasket, oils, and master mix from Bio-Rad))	$3.50
Per SNP analyzed	Primer/probe pairs – two primers per SNP, one probe per allele (e.g. from IDT, sufficient for >500 samples)	$400
Instrument cost (one-time)	Droplet generator and reader (e.g. from Bio-Rad)	$89,000
